# Letter to the Editor: Bilateral Subconjunctival Hemorrhage in a 3-Year-Old Girl with Mycoplasma Pneumonia

**DOI:** 10.2174/1874364101711010322

**Published:** 2017-11-21

**Authors:** Tatsuya Mimura, Hidetaka Noma, Satoru Yamagami

**Affiliations:** 1 Department of Ophthalmology, Tokyo Women's Medical University Medical Center East, , Japan; 2 Department of Ophthalmology, University of Tokyo Graduate School of Medicine, , Japan; 3 Department of Ophthalmology, Hachioji Medical Center, Tokyo Medical University, , Japan

**Keywords:** Subconjunctival, Hemorrhage, Mycoplasma, Pneumonia, Bilateral, Antibodies

## Abstract

We report an unusual case of bilateral subconjunctival hemorrhage following mycoplasma pneumonia. A healthy 3-year-old girl developed bilateral subconjunctival hemorrhage at 4 days after the onset of fever and respiratory symptoms such as running nose, cough, and wheezing. Laboratory data were normal except for elevation of *Mycoplasma pneumoniae* antibodies. The patient was followed without treatment and the subconjunctival hemorrhage resolved in both eyes within two weeks. To the best of our knowledge, this is the first report of subconjunctival hemorrhage following mycoplasma pneumonia. Respiratory symptoms such as cough and wheezing may cause bilateral subconjunctival hemorrhage in infants.

## 1 INTRODUCTION

Mycoplasma pneumonia is a common respiratory tract infection. *Mycoplasma pneumoniae* infection is associated with extrapulmonary manifestations in up to 25% of children that are sometimes more severe and of greater clinical importance than the primary respiratory tract infection [1]. Several cases of mycoplasma pneumonia-associated mucositis and conjunctivitis have been reported in children [2-10], but subconjunctival hemorrhage caused by *Mycoplasma Pneumoniae* has never been reported before. Here, we report an extremely rare case of bilateral subconjunctival hemorrhage in a 3-year-old girl after upper respiratory tract infection with *Mycoplasma Pneumoniae*.

## 2 CASE REPORT

A 3-year-old girl was referred to our hospital with a 1-day history of painless bilateral subconjunctival hemorrhage and a 5-day history of running nose, cough, and wheezing. Mycoplasma pneumoniae antibody titer (immunoglobulins IgG and IgM) was 1:640. Mycoplasma pneumonia had been diagnosed based on serum antibody titers and she had been treated with oral azithromycin (10 mg/kg once daily) for three days at presentation. She developed pneumothorax associated with persistent cough and wheezing, so her bilateral subconjunctival hemorrhage may have been caused by coughing. The cornea, anterior chamber, iris, and lens were normal in both eyes. Funduscopy was within normal limits. There was no coagulopathy and no history of trauma or other medical problems. The patient did not have ocular pain, photophobia, itching, discharge, or headache. She was followed without treatment because her subconjunctival hemorrhages were asymptomatic Fig. (**1**). Subconjunctival hemorrhage was still detected in both eyes at one week after the onset, but complete resolution was achieved within two weeks.

## 3 DISCUSSION

Subconjunctival hemorrhage is extremely rare after *Mycoplasma pneumoniae* infection and has never been reported previously. Infectious conjunctivitis is the most frequent ocular manifestation of *Mycoplasma pneumoniae* infection [2-10], while other rare ocular manifestations include amaurosis (Cvenkel 2003) [11], optic papillitis [12], and anterior uveitis [13-16]. These manifestations may be closely related to inflammation, infection, and tissue damage caused by this mycoplasma. However, our patient did not have inflammatory or infectious conjunctivitis and her subconjunctival hemorrhage could not be explained by direct infection of the conjunctiva. Subconjunctival hemorrhage can be associated with common systemic vascular disorders such as hypertension and arteriosclerosis [17, 18], as well as with diabetes [17, 18], trauma [17, 18], acute hemorrhagic conjunctivitis, anticoagulant therapy, conjunctivochalasis [19], and wearing contact lenses [20]. Subconjunctival hemorrhage sometimes also results from prolonged coughing, vomiting, or a Valsalva maneuver [21]. Such sudden stress can induce hemorrhage in the orbit, anterior chamber, retina, or subconjunctival space [22]. Our patient developed pneumothorax associated with persistent cough and wheezing, so her bilateral subconjunctival hemorrhage may have been caused by coughing and/or the Valsalva maneuver with elevation of the blood pressure. Increased venous pressure and congestion during the Valsalva maneuver might have led to bilateral subconjunctival hemorrhage in our patient [22].

In conclusion, this is the first report of bilateral subconjunctival hemorrhage in a patient with mycoplasma pneumonia. Ophthalmologists should be aware that respiratory symptoms such as coughing and vomiting or the Valsalva maneuver can cause bilateral subconjunctival hemorrhage in infants with respiratory tract infections.

## Figures and Tables

**Fig. (1):**
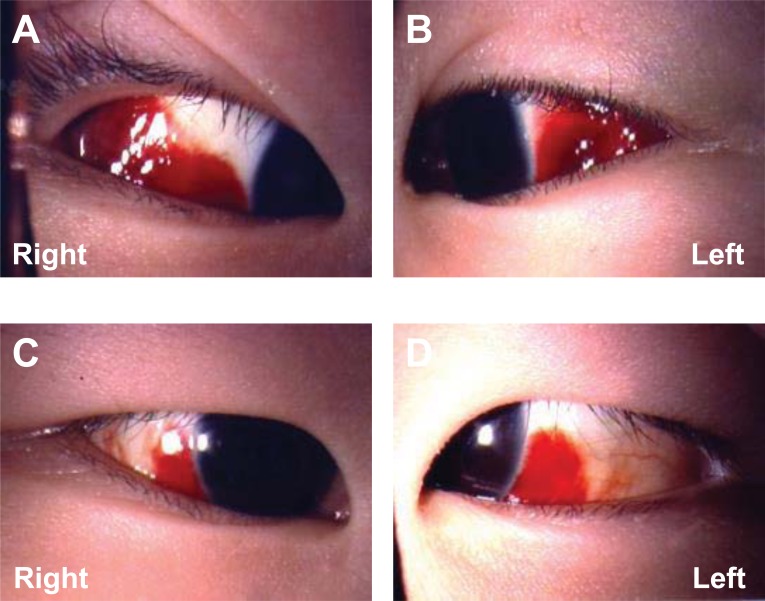
Slit-lamp photomicrographs of the anterior segment of both eyes at 1 day (A, B) and one week (C, D) after the onset of bilateral subconjunctival hemorrhage.

## PROPRIETARY INTEREST

The authors do not have any proprietary interest in this manuscript.
